# Excretion of Hexamethylenamin (Urotopin) in the Bile and Pancreatic Juice

**Published:** 1908-08

**Authors:** 


### ABSTRACT.

### Excretion of Hexamethylenamin (Urotopin) in the Bile and Pancreatic Juice.

S. J. CROWE (*Johns Hopkins Hospital Bulletin,* April, 1908) from a priori considerations concluded that urotropin, introduced as a urinary antiseptic in 1894 by Nicolaier, should when given internally be excreted in the biliary passages and there develop an antiseptic action. He accordingly undertook animal experimentation in this direction, with the following results:

1. Administered by mouth, the remedy is rapidly absorbed and remains in the circulating blood for 24 hours. Apparently the maximum concentration in the blood is reached 5 to 8 hours after administration.

2. It is excreted in the bile, pancreatic juice, and directly through the wall of the gall-bladder in dogs.

3. It was found in the saliva and milk of dogs after intravenous injection of 1 gram.

In view of these experimental findings in animals, it was determined to make a bacteriological and chemical study of the bile obtained from patients with biliary fistula, before and after giving urotropin. In four cases in which gall-bladder operations were done at the Johns Hopkins the remedy was administered, immediately afterward, in large doses (60 to 75 grains daily). The material aspirated from the sinus before the administration of the drug contained large amounts of various bacteria; that aspirated from the sinus subsequent to the administration of the medicament was entirely free from bacteria, and chemical tests showed the presence of large quantities of urotropin or its decomposition product formaldehyde. In the case of the typhoid bacillus, the rapid disappearance of the organisms was especially evident.

In a case of acute gonorrheal arthritis the medicament was also given, and some hours later the joint was aspirated and pus withdrawn which, upon chemical test, showed the presence of hexamethylenamin in considerable amount. Cultures proved that the infecting organism was the gonococcus. The dose was raised to 80 grains a day, and four days later cultures from the joint showed a marked decrease in the number of organisms. A third aspiration showed that the coccus had completely disappeared. During this period the clinical condition of the joint improved markedly. The effusion and the acute tenderness rapidly disappeared, but there still remained some periarticular infiltration and limitation of motion. After getting 80 grains daily for 24 days, the patient developed painful and frequent micturition, which however immediately ceased on withdrawal of the drug.

From these clinical observations Crowe adds the following conclusions to those he drew from his animal experiments: the remedy has been demonstrated in the bile, cerebrospinal fluid, synovial fluid, pleural effusion and blood of man. When given in sufficiently large doses (75 grains per diem) it appears in the bile in quantities which suffice to exercise a decided bactericidal action.

The remedy is probably of efficiency in:

1. Acute infections of the gall-bladder.

2. Convalescence from typhoid fever, as a prophylactic of subsequent gallstone formation and to sterilise the gall-bladder and thus prevent the patient's becoming a chronic bacillus carrier.

3. Before gall-bladder operations, as a prophylactic.

